# Supplementary figures and images for: Bridging solvent molecules mediate RNase A – Ligand binding
Source: PLoS One. 2019 Oct 23;14(10):e0224271. doi: 10.1371/journal.pone.0224271 (PMC6808499; doi:10.1371/journal.pone.0224271)

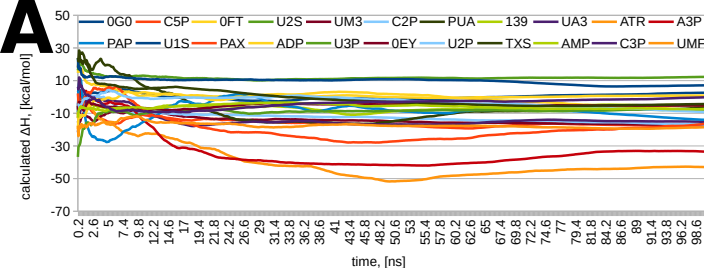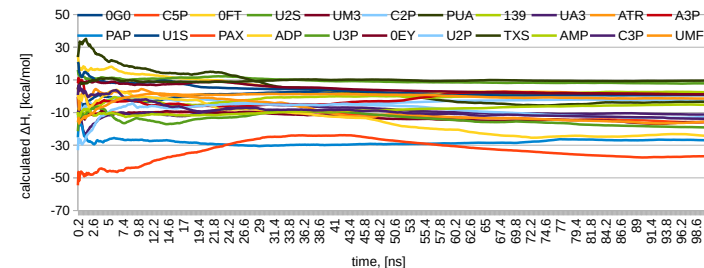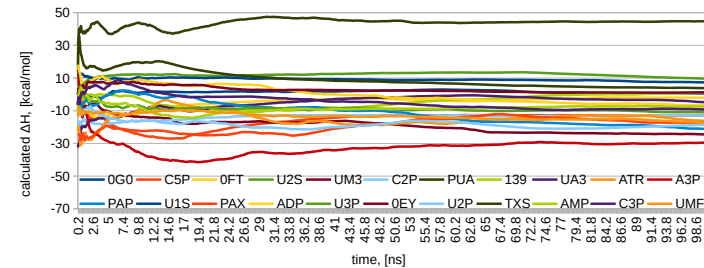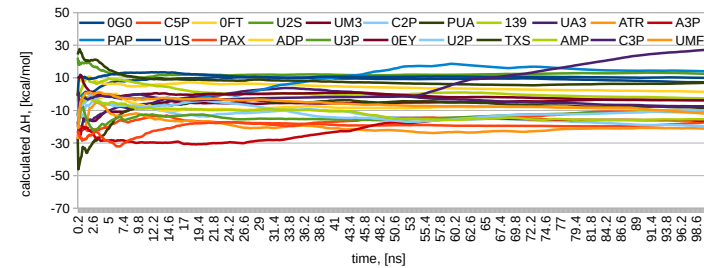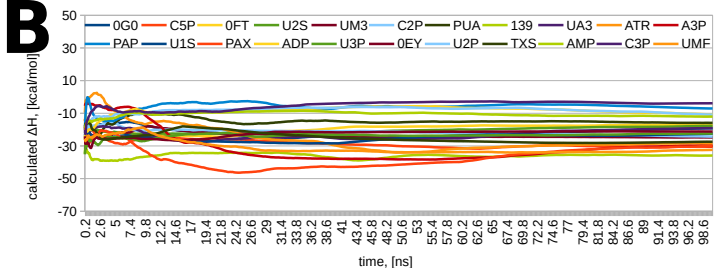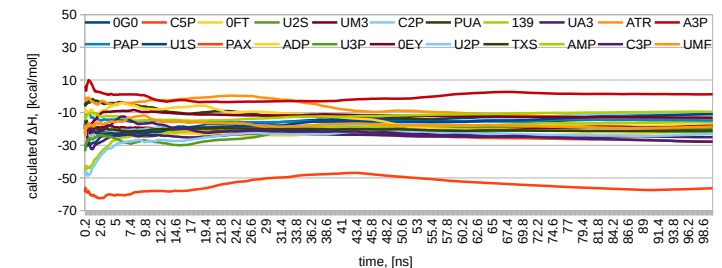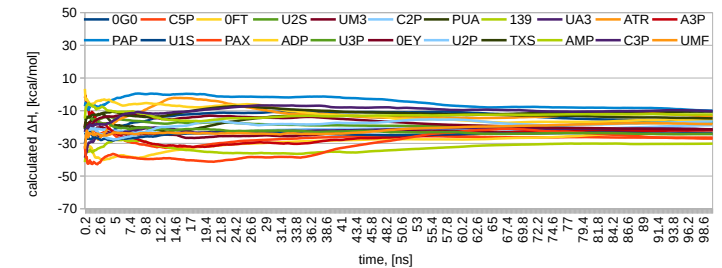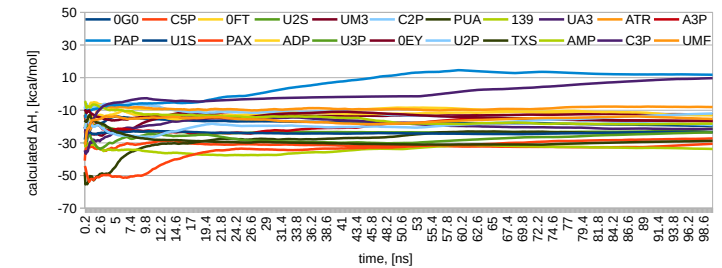

Supplement: S4 Fig — (A) Enthalpy (ΔH) convergence from the MM-PBSA calculations from replica 1 (top), replica 2, replica 3, and replica 4 (bottom); bridging waters and ions are not included in the calculations. (B) Enthalpy (ΔH) convergence from the MM-GBSA calculations from replica 1 (top), replica 2, replica 3, and replica 4 (bottom); bridging waters and ions are not included in the calculations. (PDF) [file pone.0224271.s004.pdf]
